# Foot-and-mouth disease reproduction number: a scoping review

**DOI:** 10.3389/fvets.2025.1576974

**Published:** 2025-06-05

**Authors:** Umanga Gunasekera, Kimberly VanderWaal, Jonathan Arzt, Andres Perez

**Affiliations:** ^1^Department of Veterinary Population Medicine, College of Veterinary Medicine, University of Minnesota, St Paul, MN, United States; ^2^Foreign Animal Disease Research Unit, Agricultural Research Service, U.S. Department of Agriculture, Plum Island Animal Disease Center, National Bio and Agro-Defense Facility, Greenport, NY, United States

**Keywords:** foot and mouth disease, reproductive number, endemic, epidemic, epidemiology

## Abstract

**Introduction:**

Approximately two-thirds of the countries worldwide are considered to be foot and-mouth disease (FMD) infected according to the World Organization for Animal Health (WOAH). The effective reproduction number (Re) is an important indicator to assess disease spread and evaluate the impact of preventive and control measures for FMD and other infectious diseases. Re is defined as the number of secondary infections caused by one infected animal in a susceptible population, accounting for maternal immunity, immunity from previous infections, and vaccination. When estimated at the farm/ herd level or above, this parameter is identified with terms such as Rh or Rf (commonly identified as R in this study).

**Methods:**

This study reviews the values of R estimated for FMD globally using empirical data at the farm/herd level or above. The population, intervention, comparison, and outcome (PICO) criteria were used to search different databases and to identify relevant studies, resulting in the identification of 10 peer-reviewed articles from eight different countries within the past 20 years (1994–2024).

**Results and discussion:**

Regardless of the diversity of epidemiological scenarios, the R-value of FMD remained from 0 to 13.3 with a median value of 1.68 for above farm level transmission. Results here summarize the expected range of values for R under different epidemiological conditions, contributing to the design and evaluation of prevention and control strategies and, ultimately, mitigating the impact of one of the most impactful livestock diseases worldwide. This review highlights the necessity of further studies due to a limited number of studies calculating R for FMD using empirical data.

## Introduction

Foot-and-mouth disease (FMD) is a contagious viral disease that directly or indirectly influences the dynamics of the livestock industry worldwide. The disease produces clinical signs that lead to direct economic losses for the farmers and, indirectly, disrupt international trade. FMD-affected countries are spread across many parts of the world, mainly in Asia and Africa.

The average number of secondary infections caused by one typical infectious individual in a fully susceptible fixed population during its entire infectious period is known as R0 (1). A given population is not fully susceptible to an infectious disease due to maternal immunity, immunity from previous infections, and immunity from vaccination. The effective reproductive number (Re) is calculated by accounting for such variables (2, 3). For FMD, the effective reproduction number (Re) is more appropriate for countries where the disease is endemic, while the basic reproduction number (R0) is better suited for disease-free countries. When we consider the animal population level, the Re and R0 are modified to the number of secondarily infected farms, counties, and districts from a single farm/county/district. Various terms, such as Rh and Rf, have been used to identify this parameter. For simplicity, throughout this manuscript, we will use the term R to collectively refer to indicators such as R0 and Re at the farm level, herd level, or above.

Different methods, such as those based on compartmental models, have been used to calculate R in both FMD-endemic and epidemic countries, considering country-specific scenarios using real outbreak data (4–6) and simulated models (7–9) to understand the disease transmission process. The obtained values change with the unit of analysis, species, and country (10). Knowledge of the R-value is important to decide on control measures for FMD, such as vaccination, movement control, and culling, which are considered collectively with country status, cost-effectiveness of control measures, and other disease control standards.

Objectives of this scoping review (11) are to summarize studies on the reproductive number of FMD at the between-farm/herd or higher levels using empirical outbreak data, in both endemic and epidemic settings. The results reported here will contribute to summarizing the current status of knowledge on this subject, provide a reference for parameterization of epidemiological models for the disease, and, ultimately, contribute to supporting research intended to design and evaluate prevention and control measures for FMD.

## Methods

The review question followed the population, intervention, comparison, and outcome (PICO) criteria (12). The population considered in this study is livestock (e.g., cattle, small ruminants, pigs, and buffalo). The considered intervention was calculating R, and different R calculation measures were compared to address the following questions: (a) The type of data used in the study (simulated vs. empirical), (b) What is the FMD status of the country (endemic vs. epidemic)? (c)What is the unit and the level of analysis (within farm/herd vs. beyond farm/herd level)? and (d) What other metadata are available (temporal and spatial resolution, impacts of control measures on R, FMD serotype, affected species, analysis method, assumptions, and limitations)?

To avoid selection bias in this review, we followed the PRISMA NMA Checklist criteria to search different databases to find relevant literature. R-values estimated for FMD using empirical data were assessed at the farm or herd level, and above. Experimental and simulated studies that may/may not have been parametrized based on real outbreak data to calculate R for FMD were not considered in this study. Because the objective of the study was focused on between-farm transmission, studies intended to estimate the within-farm reproduction number were not included in the review.

To assess the last 20 years’ data, online databases (PubMed, Science Direct, CAB Direct, and Ovid Medline^®^) were used to identify papers published between 1994 and 2024. Title and abstract screening were carried out in different databases for the terms “foot and mouth disease,” and at least one of the following: “basic reproduction number,” “effective reproductive number,” and “reproduction number.” Inclusion criteria for articles were: does the abstract refer to primary research, did the study mention R0 or Re of FMD, and studies in the English language. The exclusion criteria were if there were insufficient data to ensure inclusion criteria were met, an updated/withdrawn preprint, or a conference procedure only.

Following the WOAH Terrestrial Animal Code guidelines, FMD-free countries are identified as free with vaccination and free where vaccination is not practiced. Field estimates of R were defined as taking place either during an epidemic (in the case of FMD-free countries with or without vaccination) or in FMD-endemic countries. The WOAH list of members and zones recognized as free from FMD provides information regarding the current country’s status. A specific outbreak definition was not accounted as an inclusion criterion in this study, as each documented study reported different definitions of outbreaks based on relevance.

The first author carried out the literature search and data extraction. Data were extracted to a Microsoft Excel spreadsheet including details of the study (name, author, year of study, country), background information of the study (population level, endemic or epidemic country, vaccination status, serotype), and the analysis (type of data used in the analysis, R-value, methods, and limitations, validation of methods). The Grubb test was used to detect extreme R-values (13). Once outliers were removed, the median of the rest of the values was calculated to obtain a representative R-value.

## Results and discussion

**Figure 1:**
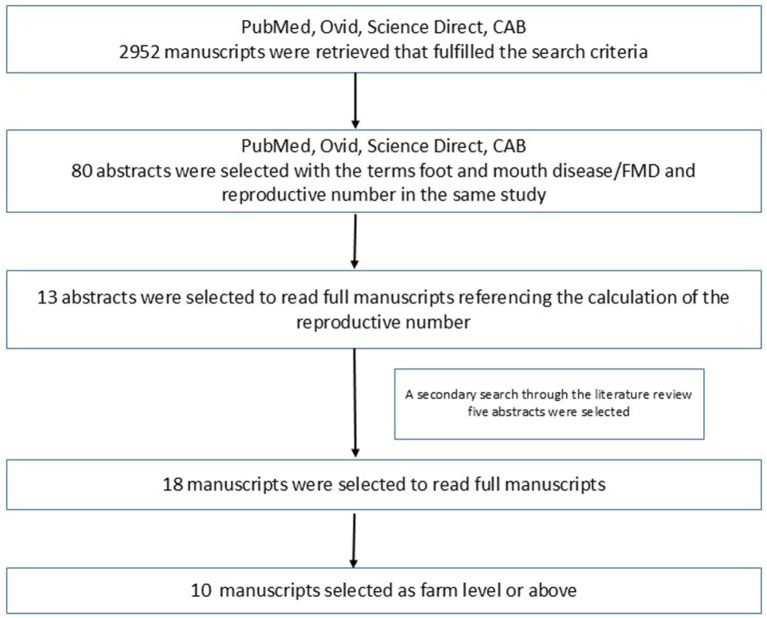
Criteria used to select articles included in this review, starting from different databases, to secondary literature search, and manual selection. Of the fully read 18 manuscripts, 10 were identified as farm-level or above using empirical data.

In PubMed, the Mesh terms “foot-and-mouth disease”[Mesh] AND “foot-and-mouth disease virus”[Mesh] AND “reproduction number” provided eight results, and four were relevant. From Ovid Medline, eight articles were identified, and five were relevant for the same search terms. Science Direct produced 1,597 results in, for the terms “foot and mouth disease” AND “reproduction number” 2000–2024, 64 articles with four relevant abstracts. From CAB abstracts, 1,339 results for [All: foot-and-mouth disease] AND [All: reproductive number], none were found to be relevant. Figure 1 summarizes the article selection process. A secondary search was carried out for abstracts from the references of the selected articles for the same terms. Five such articles were found. Full-text reading was carried out for these 18 articles. Seven of those were studies conducted at the farm level, and one study was a simulated study that was conducted between farms. These studies were excluded. The remaining 10 studies were considered in this review. These publications describe studies carried out in eight countries where R for FMD was calculated as a primary or secondary outcome using empirical data considering farm/herd level or higher.

**Figure 2:**
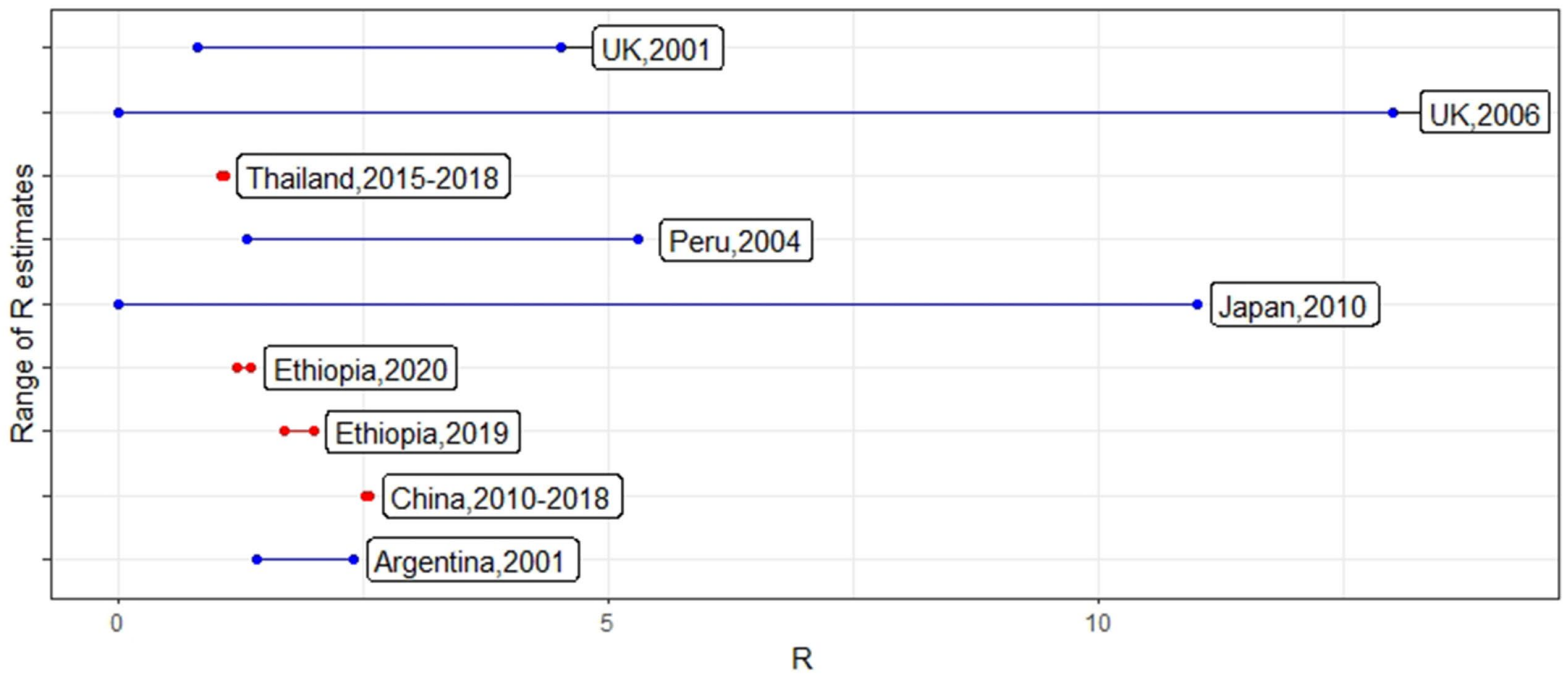
R-values reported from different countries using foot-and-mouth disease (FMD) outbreak data conducted on different periods. The line color indicates whether the country was considered to be FMD-free (blue) or endemic (red) at the time of the outbreaks.

The number of studies that focused on between-herd or above R calculation for the past 20-year period was limited to 10 studies. Chowel et al. (14) R-value (Ri 1–87.2) was identified as an outlier when compared to the other studies from the Grubbs test. R-values obtained for the rest of the studies from different countries with the considered period are shown in Figure 2.

Five studies reported results from FMD epidemics in free countries (R-values: 0–87.2) and five studies reported results from FMD-endemic countries (R-values: 1.04–5.3). Some studies calculated R0 for the whole country, while other studies selected a part of the country based on outbreak occurrence. Not surprisingly, the R-value estimated in the studies assessed here was higher in countries considered to be FMD-free compared to the endemic countries. The difference in R in epidemic vs. endemic countries was mainly due to the phenomenon that FMD would be a new disease once introduced to a country without pre-existing immunity and would therefore spread rapidly compared to the usual low R at an endemic level. FMD endemic countries at the population level showed R-values closer to one (15–18). Except for the high variability estimated in Uruguay, among the rest of the studies, the parameter fluctuated within a range of 0 and 13 (median value of 1.68), considering the differences in methods used, control measures, the time taken to detect clinical signs, and epidemic vs. endemic status. These between-farm transmission R-values are comparatively smaller compared to within-farm R-values estimated using empirical data (5, 6).

Supplementary Table 1 displays the summary of the reviewed studies (14–23). Some studies focused on modeling different species together (20), whereas others focused on one species only (16, 17, 21). The direct method of R calculation based on transmissibility, contact rate, and the duration of infectiousness (24) is usually not used with empirical data. This is because direct transmission can be accounted for only in experimental studies, which were not accounted for in this review.

Ferguson et al. (20) considered a database covering the whole country using reported outbreak data from the 2001 UK FMD epidemic to calculate two different R-values (R and R0^1^) based on the relative infectiousness of a farm. R (0.8–3.8) is the effective reproduction number, and R0^1^ (0.8–4) is the intervention-adjusted R-value. Cattle, sheep, and pig farms were considered the unit of analysis. This study modeled the 2001 UK FMD outbreak at the country level, considering the susceptible and infected compartments, accounting for the spatial locations of farms in a pair-based model (20). A pair-based approximation model considers pairs of individuals and accounts for the non-random spatial mixing of a considered population. This model comprises a system of ordinary differential equations called pair equations. According to this model, each individual (farms/animals) interacts with a limited number of spatial neighbors following a lattice model (25) to an extent determined by a spatial kernel. It is identified that pair-based models perform better compared to the individual compartmental models (26). The accuracy of the model is tested by comparing it to reported outbreak data.

Kao et al. (21) created networks of weakly and strongly connected sheep movement, considering real-time sheep movement data for the whole country to simulate an FMD outbreak using parameters mimicking the 2001 UK FMD outbreak, and compared the results with reported outbreak data. The infectious network is created by considering a fixed time frame and a directed network, removing the nodes that have the probability of being uninfected. These networks were used to calculate R0. Here, R (0–13) was calculated as a proportion between strongly connected and weaker networks. R-value changes as a combination of the infectious period and the transmission probability of nodes. R is defined as an increase in the average number of connections per node compared to a threshold of finite connections in an infinite infectious network (21). Even though this model uses a livestock movement network for R-value calculation, the calculated R-value is validated with reported outbreak data from the 2001 UK epidemic. Connections created by animal movement are considered instead of the spatial aspect.

Nishura et al. (22) described an epidemic in the Miyazaki prefecture of Japan from March 2010 to July 2010. The unit of analysis was both pig and cattle farms. Between-farm transmission of pig and cattle herds was considered separately with laboratory-confirmed outbreaks. The R-value calculated ranged from 0 to 11 as a function of calendar time. The effective reproductive number was calculated using a renewal equation. With the renewal equation, the secondary transmission rate is considered a function of time to calculate the effective reproductive number. Since this is a time-dependent method, generation time is considered as a density function in the calculation. The equation is extended to cattle and swine species. In this study, under-reporting and late reporting of outbreaks were identified as limitations (22).

Three studies used compartmental models (14, 17, 18). Chowell et al. (14) calculated R0 within and across the county level for the whole country of Uruguay in 2001. The unit of analysis was the number of infected cattle farms per county. Here, the average intra-county reproductive number was 87, accounting for homogenous mixing within the county, and the regional average (inter-county) was 0.82, accounting for spatial heterogeneity. In compartmental models, in their simplest form, when the average time to identify an infected farm is 1/ ∝ , ∝ being the infectious period, and the transmission rate is *β*, the basic reproductive ratio is calculated as


$$R_{0}=\frac{\beta }{\propto }$$

This study emphasized accounting for intrinsic characteristics of the disease, such as spatial cluster effect and seasonality, to make better predictions compared to those that did not (14). When the scale of the population level increases, such as from the farm level to the district level, the compartmental models provide better parameter values accounting for the emerging collective behavior of the disease, which can be lost at the individual level (27).

Ren et al. (17) considered pig farms in China, assuming a uniform distribution of the pig farms, connected as a network where farms are nodes in the network and edges are transactions among the farms forming a random network. A compartmental model was applied to total pig farms to obtain the value of R0 (2.5). The model is validated using least square fitting and correlation by comparing to reported FMD outbreak numbers in the years 2010 to 2018.

Tadesse et al. (18) considered two districts in Northwestern Ethiopia, with two different management systems, namely, the crop-livestock mixed farming system and commercial farms, from September 2017 to May 2018. An epidemiological unit was defined as each district due to restricted animal movement between the two districts. Susceptible, infected, and recovered numbers of animals were recorded in each farm in each district and used to first calculate the transmission parameter using a generalized linear model. The R0 was calculated from the transmission parameter considering animal-to-animal transmissions within each district. R-value was calculated separately for crop livestock mixed system (R-value = 1.68) and commercial dairy farms (R-value = 1.98). A specific validation method for R0 estimation has not been mentioned.

A study in Ethiopia (16) considered one region named Amhara for the analysis. The management system in the region was extensive. To calculate R0, as the unit of analysis, they considered age-stratified serology data of cattle from a selected few representative districts. The maximum likelihood method was used to calculate the R-value of 1.27. In this study, the R0 estimate method is not validated. The SIR model implemented using R0 for transmission rate is validated with the reported outbreak data information obtained from the farmers via a questionnaire.

Epidemic doubling time was used in studies conducted in Peru, Argentina, and Thailand (15, 19, 23). Perez et al. (23) considered the FMD epidemic in Argentina from the year 2000–2002 to calculate R_H_ for the whole country. An outbreak was defined as a herd in which an FMD virus infection was recognized officially. R_H_ accounted for vaccination and animal movement restrictions. Calculated R_H_ value ranged from 2.4 to 1.4. Using a similar method (19), in Peru considered the whole country’s outbreak reports were considered during the year 2004. The calculated R-value ranges from 1.31 to 5.3. The unit of analysis was infected cattle herds with at least one infected animal. Arjukumpa et al., calculated Rsd (1.04–1.07), at the sub-district level in northern Thailand (15). The unit of analysis was based on sub-districts with outbreak episodes between the years 2015 to 2017. If the epidemic is growing at a constant rate, the doubling time also remains constant. Doubling time increases when the epidemic is in the declining phase. Case reporting dates vary substantially from the date of onset, and the duration of infectiousness *D* is set to most likely, maximum, and minimum values. Considering homogenous mixing and the exponential growth of the epidemic, the doubling time is shown below:


$$R_{h}=1+\frac{D}{t_{d}}\;\ln_{2}$$

- D: Duration of infectiousness.
-
$t_{d}$ : Epidemic doubling time for reported outbreaks at farms (t_2_–t_1_).

The spatial distribution of outbreaks was considered using Ripley’s (k) function. Limitation of outbreak under-reporting was identified in Arjukumpa et al. (15).

R-value is an indicator of the rate at which an outbreak is expanding. Decision on control measures during a major FMD outbreak is complex, and several additional variables need to be considered along with the R-value. FMD-free countries have opted out for depopulation (20, 22) and emergency vaccination (28) in such a situation. Since such outbreaks are rare, it is beneficial to assess R from past episodes for future preparedness. For countries that are free from FMD with vaccination, monitoring the R-value during outbreak occurrences is important to evaluate the impact of control measures and to reaffirm the FMD-free status (23). In FMD-endemic countries, R-values above 1 are an indicator of the dissemination of an outbreak and help to determine if and when targeted interventions are necessary, such as vaccination, movement control, and quarantine.

In total, four studies considered vaccination when calculating R (14, 17, 22, 23). In FMD-endemic countries, both biannual and ring vaccination are practiced for FMD control (3). Typical FMDV vaccines are inactivated products that reduce clinical signs, but do not prevent infection (29, 30). In a field study conducted in Argentina during an outbreak (a country free from FMD with vaccination), it was identified that vaccination reduced the transmission coefficient (31). Reduced FMD transmission in different species of vaccinated groups, and therefore, R was confirmed in an experimental study (32).

There was not enough evidence from this literature review to determine whether the R-value would change for different animal species/different serotypes of FMD or whether it is the same value across species/serotypes during an outbreak. Most studies reported results of outbreaks caused by serotype O FMD virus. Among the considered studies, most considered the spatial aspect of the disease. Ren et al. assumed homogenous and non-spatial models that are relatively straightforward to calculate R0 compared to when accounting for population and spatial heterogeneity (27).

From this literature review, it was evident that some studies validated their methods using reported outbreak data and other methods (4/10). Three studies identified under-reporting of outbreaks as a limitation (3/10). R-value calculations are estimated to be significantly biased by outbreak reporting (33). Even when applied to the same data set at the same unit of analysis, the value calculated for R will vary (ex, UK 2001 FMD outbreak data). The limitations in each method used for R-value calculations are listed in Supplementary Table 1. Simulated studies (7–9) have proposed how transmission models can be modified to show the disease’s intrinsic characteristics, such as carrier status, seasonality, and environmental transmission.

## Conclusion

The objective of the R-value is to get an insight into how FMD transmits from one herd/farm to another. Except for Uruguay, the range of R-value was 0–13 with a median of 1.68 despite disparate epidemiological scenarios. This emphasizes the limited between-farm transmission identified based on reported outbreak data. Mathematical models aim to simulate scenarios as close as possible to the unknown reality. Noteworthy, these are average estimates and may be associated with features of the disease that, on average, remain constant among settings at the initial phase of an outbreak. Measures taken in the early phase of an outbreak have a significant impact on controlling the magnitude of an outbreak (34). Perception of R-value between farm levels or above at the early phase of an outbreak will allow policymakers to maximize the utilization of limited resources and reduce the impact of an FMD outbreak. The limited number of studies that calculated R for FMD during the recent past highlights the necessity of further studies.

## Data Availability

The original contributions presented in the study are included in the article/Supplementary material, further inquiries can be directed to the corresponding author.
